# Imputation reliability on DNA biallelic markers for drug metabolism studies

**DOI:** 10.1186/1471-2105-13-S14-S7

**Published:** 2012-09-07

**Authors:** Vladan Mijatovic, Ilaria Iacobucci, Marco Sazzini, Luciano Xumerle, Antonio Mori, Pier Franco Pignatti, Giovanni Martinelli, Giovanni Malerba

**Affiliations:** 1 Dep. of Life and Reproductions Sciences, Sec. of Biology and Genetics - University of Verona, strada Le grazie 8, 37134 Verona - Italy; 2 Dep. of Hematology and Oncological Sciences - University of Bologna, Via Massarenti, 9 40138 Bologna, Italy; 3 Dep. of Experimental Evolutionary Biology - University of Bologna, via Selmi 3, 40126 Bologna, Italy

## Abstract

**Background:**

Imputation is a statistical process used to predict genotypes of loci not directly assayed in a sample of individuals. Our goal is to measure the performance of imputation in predicting the genotype of the best known gene polymorphisms involved in drug metabolism using a common SNP array genotyping platform generally exploited in genome wide association studies.

**Methods:**

Thirty-nine (39) individuals were genotyped with both Affymetrix Genome Wide Human SNP 6.0 (AFFY) and Affymetrix DMET Plus (DMET) platforms. AFFY and DMET contain nearly 900000 and 1931 markers respectively. We used a 1000 Genomes Pilot + HapMap 3 reference panel. Imputation was performed using the computer program Impute, version 2. SNPs contained in DMET, but not imputed, were analysed studying markers around their chromosome regions. The efficacy of the imputation was measured evaluating the number of successfully imputed SNPs (SSNPs).

**Results:**

The imputation predicted the genotypes of 654 SNPs not present in the AFFY array, but contained in the DMET array. Approximately 1000 SNPs were not annotated in the reference panel and therefore they could not be directly imputed. After testing three different imputed genotype calling threshold (IGCT), we observed that imputation performs at its best for IGCT value equal to 50%, with rate of SSNPs (MAF > 0.05) equal to 85%.

**Conclusions:**

Most of the genes involved in drug metabolism can be imputed with high efficacy using standard genome-wide genotyping platforms and imputing procedures.

## Introduction

The therapeutic efficacy of any given drug is influenced by a number of different variable factors including age, weight, concurrent drug use and fixed parameters, such as gender and genetic variation as well. Many of the enzymes involved in drug metabolism are genetically polymorphic. Consequently, their activity may differ depending upon an individual's genotype [1] and hence the genotype may influence the success of individual treatment response. A well-known example comes from the warfarin, that is an effective commonly prescribed anticoagulant, for which a variant in CYP4F2 gene (a drug metabolizing enzyme) explained ~8% of dosing variability in several patient populations [2].

Imputation can be used as a part of a genome-wide association (GWA) studies or for a fine-mapping. In a genome-wide association study (GWAS) a dense set of single-nucleotide polymorphisms (SNPs) across the genome is genotyped to survey the most common genetic variation for a role in disease or to identify the heritable quantitative traits that are risk factors for disease [3] and often the individuals studied undergo drug treatment. Moreover, several, studies as in the case of our laboratory for Acute Myeloid Leukemia (AML) patients, searched for factors involved in the drug response at the genome level. The first candidate genes are the ones that are already well known [4] to be associated to the variability of drug response. Therefore it would be useful to known whether the high density arrays used to scan the genome are able to target these gene polymorphisms. Our goal is to measure the performance of imputation in predicting the genotype of the untyped SNPs using two commercial SNPs array platforms (The DMET™ Plus GeneChip array and Genome Wide Human SNP 6.0 array). The two platforms were developed with different intents: the first contains known pharmacogenetic markers for drug metabolism studies whereas the second is designed for genomic studies.

## Methods

### Subjects

Blood samples from thirtynine patients with Acute Myeloid Leukemia (AML) were collected by professor Giovanni Martinelli (Dep. of Hematology and Oncological Sciences "L. and A. Seràgnoli" - University of Bologna). Genomic DNA was extracted from peripheral blood samples with standard methods. A written informed consent was collected from each individual and the study was designed according to the ethical principles for medical research involving human subjects stated by the World Medical Association Declaration of Helsinki. The study was also approved by the Department of Hematology and Oncological Sciences "L. and A. Seràgnoli". The individuals were genotyped with the following two platforms: DMET™ Plus GeneChip array (DMET) and Genome Wide Human SNP 6.0 array (AFFY, Affymetrix Inc, Santa Clara, CA). A brief description follows.

### Genotyping

#### DMET Plus

The DMET™ Plus GeneChip array (Affymetrix Inc, Santa Clara, CA) contains 1931 SNPs and five Copy Number Variants (CNVs) distributed on 225 drug metabolizing enzymes and transporters genes. Amplified and non-amplified DNA samples were combined for the annealing and amplification steps, in which molecular inversion probes (MIP) technology was exploited to genotype all the genomic sites of interest in a single reaction. DNA samples were subsequently purified, fragmented, labeled and hybridized to the array to be scanned with the Gene Chip Scanner 3000 (Affymetrix Inc, Santa Clara, CA).

DMET Console version 1.1 (Affymetrix Inc, Santa Clara, CA) was used to perform genotype calls using standard parameters.

#### Affymetrix Genome Wide Human SNP 6.0

DNA samples were genotyped using the Genome Wide Human SNP 6.0 array (Affymetrix Inc, Santa Clara, CA), according to the manufacturer's instructions and retrieving genotype information for ~ 906,000 loci. Genomic DNA samples were firstly digested with Nsp I and Sty I restriction enzymes and then adaptor-ligated and PCR amplified using a primer that recognizes the adaptor sequence. PCR products were subsequently purified, fragmented, labeled, denatured and hybridized to oligonucleotide probes attached to the surface of the array, followed by washing and staining procedures, as well as by scanning by means of the Gene Chip Scanner 3000 (Affymetrix Inc, Santa Clara, CA). Genotyping Console 3.0 package was used to perform genotype calls using standard parameters.

#### SNPs in the study

The study was performed on 1860 of the 1931 markers of DMET because 71 markers were discarded for the following reasons:

• 13 markers have been coded in PharmGKB [4], but have not been yet validated in dbsnp[5] and therefore they do not have an adequate coding to be recognized in the reference panel, if present;

• 5 markers have two different annotated positions;

• 2 markers were duplicated;

• 5 markers presented 3 alleles in the study sample;

• 46 markers mapped on chromosome X (the software IMPUTE 2 handles autosomal markers only).

#### Quality control

Before proceeding to the analysis, we performed some quality control checks on the data. First, we tested the concordance between the genetic and reported sex to check for errors in labeling the samples. Second, all subjects showing a genotype call rate < 95% would have been removed. Third, SNPs mapping on the regions of interest (i.e. containing the drug metabolism genes, about 6000 SNPs) were removed when showing a Hardy-Weinberg p-value inferior to 0.00001.

#### Categories of SNPs

The DMET SNPs investigated were grouped into 3 classes according to their presence in the AFFY platform and in the reference panel, as follows:

• Shared: 205 markers present in both DMET and AFFY arrays (genotyped matching is performed between experimental genotypes).

• Reference Panel Only (RPO): 654 markers in DMET and in the reference panel but not in AFFY (genotyped matching is performed between DMET experimental and AFFY imputed genotypes).

• Neither in AFFY nor in reference panel (NAR): 1001 markers in the DMET, but not in the AFFY or in reference panel. Therefore, we did not perform the imputation for this group of SNPs.

Regardless of the SNP classes (Shared, RPO and NAR), markers were subdivided according to their minor allele frequency (MAF). We used the following 7 ranges: 0, 0-0.05, 0.05-0.10, 0.1-0.2, 0.2-0.3, 0.3-0.4 and 0.4-0.5.

#### Imputation

Imputation is a statistical process used to predict genotypes that are not directly assayed in a sample of individuals. The term often refers to the situation in which a reference panel of individuals genotyped at a dense set of SNPs is used to impute into a study sample of individuals that have been genotyped at a subset of the SNPs [3].

Imputation was performed using the method implemented in the software IMPUTE 2 [6]. IMPUTE 2 returns the full probability distribution of the imputed genotypes at each SNP for each individual. We generated discrete imputed genotypes by accepting a call if the posterior probability for a genotype reached a pre-specified threshold or set the genotype as missing otherwise. Genotypes from AFFY here represent the study sample and the reference panel used was prepared by Marchini et al. [6] for the CEU population, including information from the *1000 Genomes Pilot and HapMap 3 *(release Jun 2010/Feb 2009) [7].

The imputation algorithm for large population genetic datasets, built starting from a model developed by Li and Stephens [8] to capture important features of the recombination process is based on Markov chain Monte Carlo (MCMC) algorithm. Imputation typically involves a reference panel genotyped at a dense set of SNPs and a study sample genotyped at a subset of these SNPs. We chose this imputation method over the others because it allows to use multiple reference panels. Imputation was performed on the untyped markers of AFFY6.0 using the CEU reference panel prepared by Marchini et al. [6], and freely downloadable.

To speed up the procedure and to reduce the computation load imputation was performed subdividing the chromosomal regions to be imputed in partially overlapping 2Mb-chunks that have been processed independently.

We defined following parameters in order to evaluate the results of the imputation:

• Discordance: the proportion of genotype calls for which the imputed genotype did not match the experimental genotype call, averaged over all SNPs.

• Successful SNPs (SSNPs): in which the imputed genotype matched the experimental genotype for at least 37 of 39 subjects (that roughly corresponds to 5% of genotype error rate).

• Genotype error rate: proportion of unmatched genotypes over the total genotypes.

The discordance was evaluated at three different imputed genotype calling threshold (IGCT) of value. An imputed genotype was called if the corresponding posterior probability estimated by the imputation software (IMPUTE 2) was higher than investigated IGCT. Imputed genotypes below the IGCT were set as no-calls (i.e. missing genotypes). The IGCT were set on 50%, 70% and 90%.

## Results

### Genotyping

Thirty-nine (39) individuals have been genotyped with both DMET and AFFY array. Imputation was performed on the untyped markers of AFFY (study sample), as described in Materials and Methods.

### DMET markers

Table 1 reports the distribution of the investigated DMET SNPs according to their observed MAF, as well as to Shared, RPO and NAR classes.

**Table 1:** DMET SNP distribution

MAF	Shared	RPO	NAR
0	36	107	952
0-0.05	29	124	31
0.05-0.10	27	77	4
0.1-0.2	29	98	5
0.2-0.3	26	97	3
0.3-0.4	25	72	2
0.4-0.5	33	79	4
tot	205	654	1001

Shared: markers genotyped by both DMET and AFFY

RPO (Reference Panel Only): Number of markers that are present in DMET but not in AFFY

NAR (Neither in AFFY nor in Reference Panel): Number of markers not contained in reference panel so these SNPs cannot be directly imputed

For instance, 36 out of 205 shared SNPs, 107 out of 654 RPO SNPs and 952 out of 1001 NAR SNPs resulted to be monomorphic in the sample of 39 individual studied

### Shared SNPs

For 7995 genotypes (205 SNPs × 39 individuals), we observed 59 no-calls for DMET and 164 for AFFY for a corresponding genotype missing rate of 0.7% and 2%, respectively, and seventy-nine (79) discordant genotypes, corresponding to a mismatch rate of approximately 0.98%. Mismatches were randomly distributed among 40 different SNPs.

### RPO SNPs

Six-hundred and fifty-four (654) SNPs were imputed because present in the reference panel, but not in AFFY. Three different IGCT were used: 50%, 70% and 90%. Table 2 reports the overall discordance, genotype error and no-call rate for each IGCT, according to MAF values. Moreover, the number of SSNPs are shown. The largest number of SSNPs was observed for IGCT of 50%, at any MAF range.

**Table 2:** Imputation on RPO SNPs

			IGCT 50%			IGCT 70%			IGCT 90%	
		**discordance**	**No-call**	**N. of SSNPs**	**discordance**	**No-call**	**N. of SSNPs**	**discordance**	**No-call**	**N. of SSNPs**

**MAF**	**#SNP**									

0	107	3,38%	0,00%	97	2,90%	1,13%	92	2,49%	2,47%	89
0-0.05	124	3,29%	0,02%	103	2,75%	1,55%	95	2,23%	4,07%	79
0.05-0.10	77	4,46%	0,13%	59	2,93%	3,06%	55	1,83%	8,09%	45
0.1-0.2	98	4,47%	0,31%	75	3,27%	3,43%	68	2,20%	9,37%	56
0.2-0.3	97	7,27%	1,11%	66	4,39%	7,43%	61	2,17%	15,25%	42
0.3-0.4	72	6,77%	1,07%	53	3,88%	7,26%	46	2,21%	14,10%	37
0.4-0.5	79	4,41%	0,26%	63	2,92%	3,60%	59	1,95%	9,22%	47

MAF: Minor Allele Frequency

#SNP: Number of SNPs

IGCT: imputed genotype calling threshold

Discordance: the proportion of genotype calls for which the imputed genotype did not match the experimental genotype call, averaged over all SNPs.

No-call: proportion of genotypes whose posterior probability did not reach a pre-specified IGCT

For instance, the first row reports the results of 107 SNPs having a MAF = 0 in the study sample for IGCT = 50%: the discordance is 3.38% and the no-call rate is 0.00%. For the IGCT = 90%, the discordance and no-call are 2.49% and 2.47%, respectively.

## Discussion

The goal of the study is to measure the performance of imputation when predicting the genotype of SNPs involved in drug metabolism using a common SNP array genotyping platform generally used for genome wide association studies. High density SNP arrays have been projected to tag the most common SNPs along the genome. About 7 of the nearly 30 millions known SNPs have a MAF > 0.05 and only 1 million is actually contained in the standard SNP arrays. SNPs contained in the arrays were generally chosen with idea to select the minimum number of SNPs able to target the most common SNPs, exploiting the degree of linkage disequilibrium among them. Thus, SNPs in the array do not necessary belong to any class of genetic variants associated with a particular function or with the variability of any trait.

Pharmacogenetics and pharmacogenomics are interested in the study of genetic variations that are associated with variable response to drugs in terms of therapeutic effect, as well as adverse effects. In oncology, for example, researchers are interested to study mutations affecting genes coding for liver enzymes responsible for drug metabolism and mutations in tumoral DNA leading to alteration in drug response. We investigated how a high density SNPs array combined with imputation methods might help in studying a large number of genetic polymorphisms involved in the drug metabolism. To do that 39 individuals with AML were genotyped with 2 different SNP arrays: the higly dense SNP AFFY with about 1 million SNPs and the DMET array containing the most known SNPs involved in the drug metabolism. Thereafter, we checked how many SNPs were in common between the 2 arrays and the number of SNPs whose genotypes could be directly imputed from AFFY to be then matched with the DMET experimental genotypes.

A satisfactory match between experimental and imputed genotypes of a given SNP means that imputation was good enough to substitute the real genotyping for that SNPs. We measured the match between experimental and imputed genotypes looking at the overall concordance for a set of SNPs and the efficacy of imputation for any single SNP.

Imputation was performed using reference panels from HapMap 3 and the novel 1000 Genomes project. We studied the genotypes of 1860 autosomal genetic variations of the DMET array. The 39 individuals resulted to be homozygotes for the most common allele for 1095 markers. Moreover 184 markers showed a MAF < 0.05. These large numbers of genetic variations at a low MAF was expected according to the MAF indicated by the manufacturer of the array and due to the fact that the DMET was projected to contain the genetic polymorphim involved in the drug metabolism, including rare variants (someone associated with strong or adverse drug reactions). For SNPs with low MAF we could not measure with certainty the efficacy of imputation because of the low number of observations. A large part of the DMET SNPs was not neither in the AFFY nor in the reference panel used for imputation. Most of them (983/1001) presented a MAF < 0.05 (952 showed a MAF = 0). The low MAF explains why they were not present in the reference panel or in the AFFY array. With the advance of the 1000 Genomes project it is likely that they will be included in the newly updated reference panels and that imputation might become accurate also for SNPs at a very low MAF.

AFFY and DMET arrays share 205 SNPs. The genotype missing calls for DMET and AFFY were 59 (~0.7%) and 164 (~2%) respectively, indicating a higher sensibility of the DMET array. Moreover, 79 genotypes were discordant (~1%). Due to the fact that both experimentally determined and imputed genotypes are called with some degree of error, we cannot know which call (if either) is correct, so we report concordance rather than accuracy. We looked if imputation may help in detecting the array in which the genotype was badly called by selecting 2 SNPs, rs2108622 (2 mismatches) and rs1060463 (1 mismatch), presenting genotype mismatches. The genotypes were imputed after masking them in the AFFY array. Imputation suggests that the 2 rs2108622 heterozygotes called by the DMET array were 2 homozygotes as indicated by the AFFY array. On the other hand imputation and DMET were concordant to call rs1060463 homozygote in contrast with the AFFY call indicating a heterozygote (data not shown). This would suggest that the genotype calling is not totally accurate for both the arrays, as expected for the AFFY. However, since imputation suffers from a degree of uncertainty, we should perform the genotyping with an independent method to estimate the error rate of the 2 platforms and confirm the efficacy of imputation in these cases.

Other 654 SNPs present in DMET, but not in AFFY 6.0, were imputed. The MAF observed for 107 SNPs was 0 and additional 124 SNPs showed a MAF < 0.05 (see Table 2). Of the remaining 373 imputed SNPs 316 (85%), 289 (77%) or 227 (60%) SNPs, according to the increasing value of the 3 IGCTs, resulted to be successful SNPs with an overall concordance ranging from 92.6% to 98%. It is noteworthy that the "no call" rate was higher for markers having a MAF between 0.2 and 0.4 at each of the 3 IGCTs indicating that uncertainty linked to genotype calls for these SNPs is higher than for the others.

We should note that in the present paper we used a CEU reference panel including a large number of SNPs and this represents a key factor for a reliable imputation. It could be argued that the accuracy of imputation also depends on the degree of LD among SNPs of the investigated region and therefore that imputation accuracy would be lower in region presenting a low LD among SNPs. However Rosenberg et al. [9] showed that imputation accuracy, when based on an external reference (as in our case study), is not correlated with LD patterns of the imputed region, but depends largely on the genetic composition (i.e. population descent) of the reference panel used. This suggests that the accuracy of imputation might be improved when using the most suitable reference panel according to the sample of individuals studied.

Imputation resulted to be a reliable method to predict and study genotype of SNPs associated to drug metabolism when individuals were genotyped with many SNPs genome wide. In a recent study we described 2 genetic variants (rs6811453 and rs1826909) of the ADH1A gene associated to drug response in AML patients [10]. These 2 genetic variants have been imputed with a concordance of 100% (39/39 individuals for both SNPs) and therefore we would have been able to detected the association between the genetic markers and drug response also using the imputed genotypes, thus corroborating the efficacy of imputation for those SNPs.

## Conclusion

This study indicates that imputation is a valid method, however it strongly depends on the array used for genotyping (that is on SNPs present in the array; different arrays have different SNPs) and on the reference panel used. Future studies will investigate how more and more detailed reference panels impact on the efficacy of imputation, with particular attention on SNPs with rare alleles.

## List of abbreviations used

AFFY: Genome Wide Human SNP 6.0 array (Affymetrix Inc, Santa Clara, CA); DMET: DMET™ Plus GeneChip array (Affymetrix Inc, Santa Clara, CA); IGCT: imputed genotype calling threshold; MAF: minor allele frequency; NAR: neither in Affy not in reference panel; RPO: reference panel only; SNP: Single nucleotide polymorphism; SSNP: successful SNP.

## Competing interests

The authors declare that they have no competing interests.

## Authors' contributions

MS and II carried out the molecular genetic studies. GMar, GMal and PFP participated in the design of the study. VM and LX performed the bioinformatics analysis. VM, GMal and AM performed the statistical analysis. GMal, GMar, LX, PFP and VM conceived of the study, and participated in its design and coordination and helped to draft the manuscript. All authors read and approved the final manuscript.
